# Management of severe acute exacerbations of COPD: an updated narrative review

**DOI:** 10.1186/s40248-018-0149-0

**Published:** 2018-10-02

**Authors:** Ernesto Crisafulli, Enric Barbeta, Antonella Ielpo, Antoni Torres

**Affiliations:** 1 Department of Medicine and Surgery, Respiratory Disease and Lung Function Unit, University of Parma, Parma, Italy; 2 Pneumology Department, Clinic Institute of Thorax, Hospital Clinic of Barcelona - Institut d’Investigacions Biomèdiques August Pi i Sunyer (IDIBAPS), University of Barcelona, Barcelona, Spain

**Keywords:** COPD, Acute exacerbation, Hospitalization, Steroids, Antibiotics, Oxygen, High flow nasal cannulae oxygen therapy, Non-invasive mechanical ventilation, Pulmonary rehabilitation

## Abstract

**Background:**

Patients with chronic obstructive pulmonary disease (COPD) may experience an acute worsening of respiratory symptoms that results in additional therapy; this event is defined as a COPD exacerbation (AECOPD). Hospitalization for AECOPD is accompanied by a rapid decline in health status with a high risk of mortality or other negative outcomes such as need for endotracheal intubation or intensive care unit (ICU) admission. Treatments for AECOPD aim to minimize the negative impact of the current exacerbation and to prevent subsequent events, such as relapse or readmission to hospital.

**Main body:**

In this narrative review, we update the scientific evidence about the in-hospital pharmacological and non-pharmacological treatments used in the management of a severe AECOPD. We review inhaled bronchodilators, steroids, and antibiotics for the pharmacological approach, and oxygen, high flow nasal cannulae (HFNC) oxygen therapy, non-invasive mechanical ventilation (NIMV) and pulmonary rehabilitation (PR) as non-pharmacological treatments. We also review some studies of non-conventional drugs that have been proposed for severe AECOPD.

**Conclusion:**

Several treatments exist for severe AECOPD patients requiring hospitalization. Some treatments such as steroids and NIMV (in patients admitted with a hypercapnic acute respiratory failure and respiratory acidosis) are supported by strong evidence of their efficacy. HFNC oxygen therapy needs further prospective studies. Although antibiotics are preferred in ICU patients, there is a lack of evidence regarding the preferred drugs and optimal duration of treatment for non-ICU patients. Early rehabilitation, if associated with standard treatment of patients, is recommended due to its feasibility and safety. There are currently few promising new drugs or new applications of existing drugs.

## Background

In the natural history of disease, patients with chronic obstructive pulmonary disease (COPD) may experience an acute worsening of the clinical condition that is described as a COPD exacerbation (AECOPD) [1]. AECOPD may be triggered by respiratory viral and bacterial infection [2]; pollution or ambient temperature may also initiate or amplify AECOPD [2]. Characterized by increased systemic inflammatory activity [3], AECOPD has a negative impact on patients’ health status and outcomes [4, 5].

## What is an AECOPD? Definitions

The absence of homogeneity in the definition of an AECOPD has opened controversial opinions in the last few years. The possibility to define in the same ways the AECOPD could have implications on decision-making, changing therapeutic interventions in clinical trials [6, 7]. In the ‘80s, although the Anthonisen criteria [8] were based on patients’ symptoms reporting an AECOPD (increase of dyspnea, sputum purulence and sputum volume), the presence of a change in sputum characteristics, with increased cough and wheeze, could identify an AECOPD needing a specific antibiotic approach. From Anthonisen criteria, several definitions have been proposed, but in general they are based on changes in patient symptoms (dyspnea, cough, sputum) or on healthcare resources requirement (drugs, hospitalisation), or on combinations of both [6, 7]. In this context, the three recent documents related to experts consensus about management of AECOPD (the Global Initiative for Chronic Obstructive Lung Disease-GOLD document [1], the National Institute of Health and Clinical Excellence-NICE guidelines [9] and the European Respiratory Society/American Thoracic Society ERS/ATS guidelines [10]) describe definitions of AECOPD based on onset of symptoms and need of additional therapy (Table 1). Although these definitions may be usefull in the clinical practice, this approach may be too pragmatic, oversimplifing the pathogenic pathways implicated [7]. Very recently, in a holistic approach, more precise definitions of AECOPD have been considered to include not only clinical variables [7, 11, 12]; this approach may be more accurate for the differential diagnosis with other respiratory diseases.

**Table 1:** Definitions of AECOPD according to the GOLD document, NICE guidelines and ERS/ATS guidelines

GOLD document	Acute worsening of respiratory symptoms that results in additional therapy.
NICE guidelines	A worsening of the patient’s symptoms from their usual stable state which is beyond normal day-to-day variations, and is acute in onset. Commonly reported symptoms are worsening breathlessness, cough, increased sputum production and change in sputum colour. The change in these symptoms often necessitates a change in medication.
ERS/ATS guidelines	Episodes of increasing respiratory symptoms, particularly dyspnea, cough and sputum production, and increased sputum purulence

Reported from references [1, 9, 10]

## When AECOPD is severe and needs a hospitalization

Although the severity of AECOPD may be classified according to the use of medications [1], in severe AECOPD that requires hospitalization the early recognition of acute respiratory failure (ARF), either non-life- or life-threatening [1, 13] (Table 2), may be crucial for correct selection of treatment. Table 3 reports potential indicators for identifying when severe AECOPD patients require hospitalization in a medical ward or in a respiratory or medical intensive care unit (ICU) [1].

**Table 2:** Severity of hospitalized AECOPD patients

Clinical scenario	Respiratory rate (breaths/minutes)	Use of accessory respiratory muscles	Change in mental status	Supplemental oxygen given via Venturi mask able to improve hypoxemia (FiO_2_%)	PCO_2_	pH
No respiratory failure	20–30	No	No	28–35	Normal	Normal
Acute respiratory failure (non-life-threatening)	> 30	Yes	No	35–40	Increased (50–60 mmHg)	Normal
Acute respiratory failure (life-threatening)	> 30	Yes	Yes	> 40 or not improved	Increased (> 60 mmHg)	≤ 7.25

Modified from references [1, 13]

Abbreviations: FiO_2_ indicates fraction of inspired oxygen, PaCO_2_, partial arterial carbon dioxide pressure

**Table 3:** Potential indicators for do-not-hospitalize AECOPD patients or hospitalization in a general ward or in a respiratory or medical intensive care units (ICU)

Variables	Do-not-hospitalize	General ward	Respiratory or medical ICU
Insufficient home support		X	
Good response to initial medical management	X		
Failed response to initial medical management		X	
Fewer symptoms (dyspnea on effort, RR < 30 breaths/min, SatO_2_ > 90%, no confusion, no drowsiness)	X		
Severe symptoms (resting dyspnea, RR ≥ 30 breaths/min, SatO_2_ ≤ 90%, confusion, drowsiness)		X	
Very severe symptoms (dyspnea) that respond inadequately to initial emergency therapy			X
Presence of serious comorbidities (e.g. heart failure, newly occurring arrhythmias, etc.)		X	
Onset of new physical signs (e.g. cyanosis, peripheral edema)		X	
Acute respiratory failure (without use of accessory respiratory muscles and change in mental status)		X	
Acute respiratory failure (with use of accessory respiratory muscles and change in mental status)			X
Persistent or worsening hypoxemia (PO_2_ < 40 mmHg) and/or severe respiratory acidosis (pH < 7.25)			X
Need for IMV			X
Hemodynamic instability (need for vasopressors)			X

Modified from report [1]. The cross mark identifies the correct setting

Abbreviations: *RR* indicates respiratory rate, *SatO*_*2*_ oxygen saturation, *PaO*_*2*_ partial arterial oxygen pressure, *IMV* invasive mechanical ventilation

## Why a severe AECOPD is important?

Hospitalization for AECOPD worsens the course of COPD disease and involves a rapid decline in health status associated with high mortality [5, 14, 15]. Although male gender has been identified as a risk factor for higher morbidity and mortality in AECOPD [14], recent data indicate that female COPD patients may have a higher number of hospitalizations with a prolonged length of stay [16]. In any case, severe AECOPD increases the possibility of subsequent COPD-related hospitalizations [5], with potential causal associations among past and future severe exacerbations [17]. This could be related also to a distinct phenotype of COPD, *the frequent exacerbator* [18], worsening the clinical outcomes [19]. In this context, a history of at least two prior incidents of severe AECOPD is associated with a new severe event (adjusted odds ratio [OR] 6.73; 95% confidence interval [CI], 3.53 to 12.83) and an increased risk of death (adjusted OR 7.63; 95% CI 3.41 to 17.05) [20]. Variables related to age, low body mass index, cardiac failure, diabetes mellitus, ischemic heart disease, malignancy, forced expiratory volume in the 1^st^ second (FEV_1_), long-term oxygen therapy, and the partial arterial oxygen pressure (PaO_2_) on admission of severe AECOPD were significantly associated with long-term mortality during a 2-year follow up period [21]. The presence of coexisting asthma has also been proved to be associated with frequent severe AECOPD [22]. In general for a severe hospitalized AECOPD, the weighted average of the in-hospital mortality, and of the mortality at 1 year and 5 years, is 6.7% (95% CI 5.7 to 7.7), 33% (95% CI 25 to 40) and 51% (95% CI 38 to 63), respectively [23]. Recent evidence about worse outcomes in hospitalized AECOPD shows links with the presence of hyponatremia [24], hypoalbuminemia [25], ischemic heart disease [26], and acute kidney injury [27]. Recently, an English study reported that patients having a pulmonary arterial enlargement, defined by a pulmonary artery to aorta (PA/A) ratio > 1 at chest CT scan, associated with an increased level of troponin (> 0.01 ng/mL), had an increased probability of having ARF, ICU admission or in-hospital mortality in comparison to patients without both these factors [28].

## Why a treatment in a severe AECOPD is important?

Treatments for AECOPD aim to minimize the negative impact of the current exacerbation and to prevent subsequent events [1], such as the relapse of AECOPD [29] and, in cases of hospitalization, reduce early readmissions to hospital [30] occurring within ≤30 days of discharge. Although there are known predictors of early readmission [31–33], re-hospitalization for a new severe event is required in 18% of AECOPD patients admitted [34] and was associated with a subsequent progressive increase in the risk of death in a long-term follow up [34]; for this reason, early readmission should be considered in these patients as a marker of more severe disease with a worse prognosis [35]. Although the initial treatment may be a useful predictor of subsequent clinical outcomes [36], treatment failure, which occurs in 14% [37] to 18% [38] of treated patients, may be predicted by a measure of daily health status (by the Clinical COPD Questionnaire-CCQ) [38]; moreover, an increased inflammatory level at admission (C-reactive protein [CRP] + 1 mg/dL OR 1.07; 95% CI 1.01 to 1.13) and the use of penicillins or cephalosporins (OR 5.63; 95% CI 1.26 to 25.07) were independently associated with an increased risk of treatment failure, whereas cough at admission (OR 0.20; 95% CI 0.05 to 0.75) was associated with a lower risk [37].

In this narrative review, based on a search on Medline completed in the month of May 2018, we update the scientific evidence about the in-hospital pharmacological and non-pharmacological treatments used in the management of a severe AECOPD. For the pharmacological approach we have divided the evidence according to the class of drugs (inhaled bronchodilators, steroids, and antibiotics). Non-pharmacological treatments include the use of oxygen, high flow nasal cannulae (HFNC) oxygen therapy, and non-invasive mechanical ventilation (NIMV) and pulmonary rehabilitation (PR). Finally, we review some studies concerning non-conventional drugs that have also been proposed for severe AECOPD.

## Bronchodilators

Although recommended with or without short-acting anticholinergics as the initial bronchodilators to treat an AECOPD, short-acting β_2_ agonists (SABA) have no high-quality evidence from randomized-control trials [1]. The use of these medications is in line with recent treatment patterns of hospitalized patients [39] in which only 5% of patients did not receive a SABA, while 72% received a single-product SABA and 46% received a combination with SABA plus a short-acting anticholinergic [39].

A Canadian meta-analysis performed in COPD patients with an acute airflow obstruction found no difference in the effect of bronchodilator delivery by a metered-dose inhaler (MDI) or wet nebulizer based on objective measurements [40]. Similarly, a recent Cochrane review comparing the effects of nebulizers versus pressurized MDI (pMDI) plus spacer or dry powder inhalers (DPI) [41] found no significant difference in the change in FEV_1_ at 1 h after dosing between nebulizers versus pMDI plus spacer although there was a trend for greater improvement in FEV_1_ when treating with nebulizers [41]. No data of sufficient quality have been published comparing nebulizers versus DPIs [41]. In conclusion, the limitations of the difficult-to-pool and low-quality data did not permit the authors to provide evidence to favor one mode of delivery over another [41].

In general, spacer devices are recommended for bronchodilator delivery with a pMDI [1]. A study evaluating the necessary inspiratory flow using different DPIs in patients admitted to hospital during an AECOPD demonstrated the ability of patients to use these inhalers [42]. At admission, the median (interquartile range) of inspiratory flow (L/minutes) using the appropriate simulators of the resistance and the percentage of patients (%) achieving the minimum necessary inspiratory flow of 30 L/min required for each DPI device were 40 L/m (30–50) (91%), 50 L/m (40–60) (95%), 60 L/m (50–87.5) (100%), and 30 L/m (20–40) (82%) for Turbuhaler®, Diskus®, Aerolizer®, and Handihaler®, respectively [42]. This might open up the possibility of using long-acting β_2_ agonists (LABA) for the treatment of AECOPD [43]. A proof of concept [44] performed in AECOPD patients having a mild-to-moderate worsening of dyspnea demonstrated that an early treatment with doubling dose of a combination of a LABA (Salmeterol) plus an inhaled corticosteroid (ICS, Fluticasone Propionate) for 10 days has a potential effect avoiding the requirement of prednisone within 30 days of the onset. In a single-center, open-label, randomized, crossover, and single-blind trial on 12 hospitalized AECOPD patients, a recent study exploring the acute effect of indacaterol found a significant mean increase in FEV_1_ and forced vital capacity (FVC), with the highest increase at the dose of 300 mg; however, transient episodes of oxygen desaturation were observed in some patients with relatively well-preserved PaO_2_ (− 5.9 mmHg [95% CI -1.4 to − 10.4] and − 6.2 mmHg [95% CI -2.7 to − 9.8] in the mean peak in PaO_2_ over 6 h in the group treated with indacaterol 150 mg and indacaterol 300 mg, respectively) [45]. Similar increases were observed when indacaterol was administered over 5 days in the emergency department in comparison to salbutamol [46].

### Main take home messages

The large use of SABA and short-acting anticholinergics during an AECOPD is a common therapeutic practice, although to date there are not solid evidences. There are also limitated data about the better bronchodilator delivery. The use of LABA during AECOPD, although limitated, could have large employ in the manteinance therapy after discharge.

### Unmet needs or future clinical research

There are not quality data about the use of long-acting muscarinic antagonists (LAMA) during a severe AECOPD. In the era of dual bronchodilation in the treatment for COPD [47] this represents a strong unmet needs that should be investigated.

## Steroids

There is strong evidence for the use of systemic steroids in severe AECOPD [10]. This has been reported in the recent European Respiratory Society/American Thoracic Society consensus guideline providing clinical recommendations for the treatment of AECOPD [10].

Several studies have demonstrated an improvement of several outcomes in AECOPD patients using systemic steroids, including length of hospital stay [48, 49], in-hospital oxygenation [48, 49], FEV_1_ [48–50], dyspnea [50], risk of relapse [50], and rate of treatment failure [49, 51]. Prednisone/prednisolone is the preferred intravenous or oral steroid [48, 50, 52–54]. The oral administration of prednisolone is as effective as intravenous administration [53]. A short duration of treatment is preferred to a long duration [52, 55]. Several studies have been performed on hospitalized AECOPD but not on ICU patients [48–50], while some have been performed in critically ill patients [51, 56].

In 1999, two studies [48, 49] performed in patients without ARF and placebo-control demonstrated the efficacy of oral prednisolone (30 mg for 14 days) [48] and intravenous methylprednisolone (500 mg for 3 days followed by oral prednisone 60 mg for 4 days with a scalar dose until 8 or 2 weeks, according to the study group) [49] at improving FEV_1_ and reducing hospital stay. For the first time in trials, the latter study [49] reported treatment failure as a primary outcome, defined by the assessment of in-hospital deaths due to any cause, the need for NIMV or ICU admission, hospital readmission, and the need for intensification of therapy. The authors demonstrated a lower rate of combined failure variables at 30 days (23% versus 33%, *p* = 0.04) and at 90 days (37% versus 48%, *p* = 0.04) in patients using steroids in comparison to placebo [49]. Finally, in a three-arm design (patients receiving 8 or 2 weeks of steroids and patients receiving placebo) it was demonstrated that the long steroid regimen of 8 weeks did not increase the clinical benefits of a short regimen of 2 weeks [49].

To study the outcomes of the risk of relapse after a severe AECOPD, Aaron and colleagues [50] enrolled AECOPD patients who were being discharged from the emergency department and treated with 40 mg of oral prednisone for 10 days. The authors found a lower rate of relapse at 30 days (27% versus 43%, *p* = 0.05) and a longer time to relapse (23 days versus 7 days, hazards ratio 0.56 [95% CI 0.32 to 0.99], *p* = 0.04) in patients with steroids versus placebo [50]. Furthermore, patients treated with prednisone showed improvements in their FEV_1_ (from day 1 to day 10) and dyspnea scores [50].

Studies performed specifically in mechanically ventilated AECOPD patients or those admitted to the ICU include the Spanish double-blind, placebo-controlled trial of Alía and colleagues [51], which was performed on 83 acidotic AECOPD patients who received intravenous methylprednisolone (from a dose of 2 mg/kg/day for 3 days to 0.5 mg/kg/day from days 7 through 10). Patients in the steroid group showed a reduction in the duration of mechanical ventilation in comparison to placebo (median [interquartile range], 3 days [2–6] versus 4 days [3–7], *p* = 0.04, respectively) [51]; however, when comparing patients with conventional ventilation (46 patients) and NIMV (37 patients) the use of steroids did not appear to reduce the duration of ventilation in comparison to the placebo. Interestingly, the rate of NIMV was lower in the steroid group (0%) in comparison to the placebo (37%). Another prospective, open-label, randomized, placebo-controlled trial on the use of oral prednisone (1 mg/kg/day for up to 10 days) performed on 217 acidotic AECOPD patients requiring initial conventional mechanical ventilation (*n* = 53) or NIMV (*n* = 164), found contrasting findings [56]. There was no difference in primary outcomes (ICU mortality, evaluated in all ventilated and in conventional and NIMV) or secondary outcomes (failure of NIMV, duration of mechanical ventilation, ICU length of stay) [56]. The different doses of steroid used in the studies [51, 56] and the severity of AECOPD (more severe respiratory acidosis and hypercapnia in the latter [56]) may explain the different results.

With regards to the route of steroid administration (parenteral versus oral), a study has demonstrated that oral administration with prednisolone (60 mg for 5 days) was no less effective than intravenous therapy [53], with no difference in treatment failure in the early phase (within 2 weeks) or at 90 days. The length of hospital stay, moreover, was similar among the oral administration (11.2 days) and the intravenous therapy group (11.9 days) [53]. A recent study [57] comparing AECOPD patients receiving a fixed, low dose of oral steroids (methylprednisolone 32 mg/day for 7 days) with patients receiving higher doses of intravenous steroids (methylprednisolone at 1 mg/kg/day for 4 days and 0.5 mg/kg/day for 3 days) demonstrated similar results between study groups in terms of lung function, symptoms and oxygenation [57]. In line with these findings about the optimal dose and route of steroid administration, a pharmacoepidemiological cohort study conducted in more than 400 US hospitals involving almost 80,000 AECOPD patients [58] demonstrated that the risk of treatment failure (initiation of mechanical ventilation, in-hospital mortality, or readmission for AECOPD within 30 days of discharge) among patients treated with low doses of steroids administered orally was not worse than for those treated with higher doses administered intravenously [58]. In the context of the optimal dose of steroids in AECOPD patients treated in the ICU, another recent pharmacoepidemiological cohort study [59] performed in 473 hospitals on more than 17,000 patients examined the effectiveness and safety of a lower dose (methylprednisolone ≤240 mg/day) versus a higher dose (methylprednisolone > 240 mg/day) of steroids [59]. The authors found that patients using lower versus higher doses of steroids had similar hospital mortality (OR 0.85; 95% CI 0.71 to 1.01; *p* = 0.06), defined as a primary outcome. However, a lower dose of steroids was associated with a reduced length of hospital and ICU stay, hospital costs, length of invasive ventilation, need for insulin therapy, and fungal infection [59].

Based on the concept that the use of systemic corticosteroids for a longer time is associated with adverse effects such as osteoporosis, hyperglycemia and muscle weakness, the noteworthy trial of Leuppi and colleagues [52] (REDUCE: Reduction in the Use of Corticosteroids in Exacerbated COPD), published in 2013, defined the optimal dose (prednisone 40 mg daily) and duration of steroid treatment (5 days) in hospitalized AECOPD. The short-term treatment of 5 days, in fact, was non-inferior to conventional treatment (14 days). The percentage of patients having a new exacerbation (median 180 days of follow up) was lower in the short-term treatment in comparison to conventional treatment (36% versus 37%, *p* = 0.006 for the intention-to-treat [ITT] analysis, and 37% versus 38%, *p* = 0.005 in the per-protocol [PP] analysis, respectively). Similarly, the duration of hospital stay was lower (median 8 days [IQR 5–11] versus 9 days [IQR 6–14]; *p* = 0.04, respectively) and the cumulative prednisone dose (difference in mean − 414 mg.; *p* < 0.001) was lower in the short-term group in comparison to the conventional group [52]. A Cochrane review, updated in 2018 [55] suggests that in a severe AECOPD the likelihood of having a worse outcome is lower in patients using an oral steroid for 5 days than with a longer course (10 to 14 days) [55]. This review [55], however, excluded studies with patients requiring assisted ventilation. In this context, a previous study performed on AECOPD requiring hospitalization but with ARF and comparing the same dose of methylprednisolone (2 mg/kg/day) but with different duration (3 or 10 days) [60] did not confirm that a short treatment is better for these patients. In fact, although both study groups (3 and 10 days) experienced improvements in oxygenation and FEV_1_, and the re-exacerbation rate at 6 months was similar, the improvements were more marked over the course of 10 days of steroids, as were those in FVC and dyspnea on exertion [60].

Although domiciliary inhaled corticosteroids do not influence the early inflammatory response and the clinical presentation of hospitalized AECOPD [61], the use of these medications during an AECOPD may be a valid alternative to systemic steroids for hospitalized patients as well; this approach might reduce the risk of side effects. A multicenter, double-blind, randomized, placebo-controlled trial performed on 199 not acidotic and not hypercapnic AECOPD patients compared nebulized budesonide (2 mg four times/day) and oral steroids (prednisolone 60 mg/day) [62]. In comparison to placebo, both treatments (budesonide and prednisolone) demonstrated an improvement in post-bronchodilator FEV_1_ and the difference between the two groups was not significant [62]. Another trial [63] performed on AECOPD patients without a severe airflow obstruction and without a ARF (hypercapnic patients with a partial arterial carbon dioxide pressure-PaCO_2_ > 70 mmHg were excluded a priori), was aimed to evaluate the clinical efficacy of comparison between nebulized budesonide (2 mg three times/day) versus intravenous methylprednisolone (40 mg/day). Both groups showed similar improvements in symptoms, pulmonary function and arterial blood gas analysis, although the incidence of adverse events was lower in the budesonide group [63].

### Main take home messages

The use of steroids during a severe AECOPD appears justified from solid evidences. This is confirmed by improvements on several in-hospital (symptoms, length of hospital stay, treatment failure) and post-discharge (risk of relapse) outcomes. The oral administration should be preferred due to the similar efficacy of intravenous and for the limitation of systemic effects.

### Unmet needs or future clinical research

In COPD patients molecular and immunological mechanisms of steroid resistance are documented [64]. To identify a cluster of steroid-responder patients could highlight a different therapeutic approach in the management of severe AECOPD.

## Antibiotics

Data coming from sputum cultures suggest that bacterial infection is a more prevalent cause of AECOPD [2, 65, 66], with a correlation between sputum color and the presence of potentially pathogenic bacteria [67, 68]. In general, a sputum color of green or yellow was most likely to yield potentially pathogenic microorganisms (59% and 45%, respectively), in comparison to clear (18%) and rust-coloured sputum (39%) [67]; sputum color predicts a positive culture [67]. In severe AECOPD patients requiring mechanical ventilation, potentially pathogenic microorganisms (PPMs) and/or a positive serology were present in 72% of patients [69]. A bronchial microbiological pattern corresponding to community-acquired pathogens (*Streptococcus pneumoniae*, *Haemophilus influenzae*, and *Moraxella catarrhalis*) was present in 56% of positive samples, while a high percentage of positive samples (44%) contained gram-negative enteric bacilli (GNEB), *Pseudomonas*, and *Stenotrophomonas spp.* [69]. Interestingly, the presence of pathogens was clinically unpredictable [69]. Being a non-current smoker, ≥ 2 AECOPD or ≥ 1 admission for AECOPD in the previous year and a CRP < 5 mg/dL were proved to be independent factors predicting the presence of microorganisms resistant to conventional treatment (MRCT) (40% of isolates in hospitalized AECOPD patients) [70]; in this context, although patients with MRCT had longer hospital stays, the mortality rate at 30 days, 1 year and 3 years was comparable to that of patients with microorganisms sensitive to conventional antimicrobial treatment (MSCT) [70]. In any case, the use of antibiotics is recommended in AECOPD with sputum purulence and especially for very severe patients [67–70].

Recently, with the aim of differentiating among chronic colonization and acute infection in AECOPD patients, levels of procalcitonin have been considered as a marker for the use of antibiotics [71, 72]. An updated Cochrane meta-analysis [73] showed that in acute respiratory tract infections the use of procalcitonin is useful to initiate or discontinue antibiotics; this results in lower risks of mortality, lower antibiotic consumption, and lower risk for antibiotic-related side effects [73]. In severe AECOPD patients with low serum procalcitonin values (< 0.1 ng/ml), treatment with antibiotics has no benefits in comparison to placebo [74].

The use of antibiotics in AECOPD without signs of infections remains controversial [1]. The recommendation regarding length of antibiotic therapy (5 to 7 days) in AECOPD [1, 75] originates from the use of levofloxacin 500 mg, which has achieved clinical and bacteriological success regardless of the therapeutic course (5 or 7 days) [75]. However, this evidence was obtained in patients being managed in the community as either general practice patients or outpatients [75] and not in hospitalized severe AECOPD patients. A Cochrane review [76] showed that in AECOPD patients who need hospital admission for severe exacerbations, antibiotics reduce treatment failures (risk ratio 0.77, 95% CI 0.65 to 0.91, quality of evidence GRADE [Grading of Recommendations, Assessment, Development and Evaluation]: high) but not the length of hospital stay or mortality (low grade of evidence) [76]. The role of antibiotics is clearer in patients admitted to the ICU [76], even if documented in only one study [77]. Antibiotics reduce treatment failures up to 4 weeks (risk ratio 0.19, 95% CI 0.08 to 0.45, quality of evidence GRADE: high), all-cause mortality (odds ratio 0.21, 95% CI 0.06 to 0.72, quality of evidence GRADE: high), and length of hospital stay (mean difference − 9.60 days; 95% CI -12.84 to − 6.36 days) [76]. The authors concluded that the benefits of antibiotics in the ICU are evident [76], while in inpatients the evidence is inconsistent and stems from old studies [78–80].

With regards to the choice of antibiotic, a randomized, placebo-controlled trial in 2010 evaluated the effects of doxycycline (200 mg) in addition to systemic corticosteroids [81]. On day 30 treatment success (the primary outcome defined as the cure or improvement of signs and/or symptoms of AECOPD, without sign or symptoms of infection) was not significant in intention-to-treat analysis (OR 1.3; 95% CI 0.8 to 2.0; *p* = 0.32) or per-protocol [81]. The clinical success at 10 days (secondary outcome) favored doxycycline (OR 1.9; 95% CI 1.1 to 3.2; *p* = 0.03) only in ITT, but not in PP.  Furthermore, at 10 days doxycycline was superior to placebo in term of change in bacteriological response, serum CRP and symptom scores, in particular cough and sputum purulence [81].

A cornerstone study about the use of antibiotics in severe AECOPD but requiring mechanical ventilation was the prospective, randomized, double-blind, placebo-controlled trial of Nouira and colleagues [77], who evaluated the effects of a fluoroquinolone, ofloxacin (400 mg for 10 days). In comparison to the placebo (*n* = 46), patients using ofloxacin (*n* = 47) had a reduction in primary outcomes, in particular percentage of deaths in the ICU (4% versus 17%, *p* = 0.05), deaths in hospital (4% versus 22%, *p* = 0.01), need for additional course of antibiotics (6% versus 35%, *p* = 0.0006), and combined events (11% versus 57%, *p* < 0.0001) [77]. Similarly, in terms of secondary outcomes ofloxacin was superior to placebo in number of days of mechanical ventilation (mean 6.4 days ±3.1 versus 10.6 days ±5.1, *p* = 0.04), duration of ICU stay (mean 9.4 days ±5.2 versus 14.5 days ±6.0, *p* = 0.02), and duration of hospital stay (mean 14.9 days ±7.4 versus 24.5 days ±8.5, *p* = 0.01) [77]. The same researchers in the same series of patients (*n* = 170) evaluated the combination of trimethoprim and sulfamethoxazole (160/800 mg twice daily for 10 days) versus ciprofloxacin (750 mg twice daily for 10 days) in a randomized, double-blind trial [82]. In this case, the number of deaths in hospital or the ICU, the need for additional antibiotics, and the combined events (considered primary outcomes) were similar between study groups, including the duration in days of mechanical ventilation, the duration of hospital or ICU stay, and the exacerbation-free interval (secondary outcomes) [82]. The authors concluded that the efficacy of trimethoprim-sulfamethoxazole was similar to that of ciprofloxacin [82].

### Main take home messages

In AECOPD with history of purulent sputum an antibiotic is suggested, although the role of this treatment in hospitalized AECOPD appears controversial and not fully clear. In patients admitted in ICU it seems have the major benefit. The levels of procalcitonin could better identify patients needing an antibiotic therapy.

### Unmet needs or future clinical research

Although GOLD guidelines recommend an antibiotic therapy for 5 to 7 days [1], there is not a strong evidence about the choice of antibiotic and the duration of treatment.

## Oxygen therapy, high flow nasal cannulae (HFNC) oxygen therapy, and non-invasive mechanical ventilation (NIMV)

The routine use of titrated oxygen treatment is recommended in hospitalized AECOPD, due to the lower risk of death and lower likelihood of respiratory acidosis or hypercapnia than in patients who received high flow oxygen [83]. Blood gases should be monitored to ensure a good level of oxygenation without carbon dioxide retention and/or worsening acidosis; the PaO_2_ should be maintained at 7.3–10 kPa (SaO_2_ 85–92%) to avoid the dangers of hypoxia and acidosis [84]. A recently developed device (FreeO_2_) automatically adjusts the oxygen flow rates based on patients’ needs, in order to limit hyperoxia and hypoxemia in hospitalized AECOPD patients [85]. In comparison to manual oxygen titration this device improved the percentage of time within the target SaO_2_ (significantly higher with FreeO_2_) and the time with severe desaturation and hyperoxia (reduced with FreeO_2_); furthermore, the length of hospital stay was lower in patients using FreeO_2_ [85].

HFNC oxygen therapy is a recent and easy therapeutic methodical, able to deliver high fraction of inspired oxygen (FiO_2_), generating a low level of positive pressure and providing a washout of nasopharyngeal dead space [86–89]. The effects of HFNC increase the end-expiratory lung volume, improve the oxygenation and the breathing pattern, reduce the work of breathing [86–89]. It is considered an alternative for patients with severe hypoxemic ARF [86], although in comparison to conventional oxygen therapy and NIMV, a recent systematic review with a meta-analysis did not show for the HFNC a significant reduction of intubation rate and mortality [90]. Similary, a Cochrane review [91], due to very limited data, is unable to demonstrate whether HFNC provides an efficacy respiratory support for adult ICU patients. In addition, some risks of bias among included studies, differences in patient groups, and high levels of statistical heterogeneity for some outcomes, lead to uncertainty results [91].

The role of HFNC in stable hypercapnic patients has been recently demonstrated as safe [92, 93]. HFNC leads to a flow-dependent reduction in hypercapnia level; this is most likely achieved by a washout of the respiratory tract and a functional reduction in dead space [93].

In 24 AECOPD hospitalized patients, a single-centre randomized-controlled cross-over trial has evaluated the short-term effects of HFNC on PaCO_2_ level [94]. Both interventions (HFNC at 35 l/min and standard oxygen delivered via nasal prongs) were administred for 30 min, with oxygen titrated to maintain the patient’s baseline saturation and with 15 min of washout between interventions. The difference in transcutaneous carbon dioxide tension (PtCO_2_) at 30 min (primary outcome) was lower for HFNC compared with standard oxygen (− 1.4 mmHg; 95% CI − 2.2 to − 0.6; *p* = 0.001). At 30 min, however, there was no difference between groups in SaO_2_ and respiratory rate [94].

In 88 severe AECOPD with moderate hypercapnic ARF the clinical effectiveness of HFNC has been evaluated in a very recent prospective observational trial in comparison to NIMV [95] and has failed to demonstrate any significative effect. The intubation rate and the 30-day mortality were similar between groups, such as pH, PaO_2_ and PaCO_2_ after 6 and 24 h [95]. However, this study reports methodological, inherent bias and tecnical (setting of NIMV) limits that probably can be explained for different levels of treatment [96].

In COPD patients, the increase in PaCO_2_ level with respiratory acidosis is associated with a worse outcome [84, 97, 98], including the risk of ICU admission [84] or death [84, 97, 98]. Several randomized trials have demonstrated the efficacy of NIMV (using bilevel positive airway pressure) in AECOPD in comparison to standard care [99–103]; for this reason data from the Healthcare Cost and Utilization Project’s Nationwide Inpatient Sample confirm that the use of this treatment has increased significantly over time among patients hospitalized for AECOPD, whereas the need for intubation and in-hospital mortality has declined [104]. The official ERS/ATS clinical practice guidelines about noninvasive ventilation for acute respiratory failure [105], providing evidence-based recommendations, report that in AECOPD patients the use of NIMV in patients with ARF leading to acute or acute-on-chronic respiratory acidosis (pH ⩽7.35) is strongly recommended (GRADE: high level of evidence), while a conditional recommendation with low certainty of evidence is reported for the prevention of acute respiratory acidosis (PaCO_2_ normal or elevated but pH normal) [105]. In the context of respiratory acidosis, NIMV is important to avoid endotracheal intubation and invasive mechanical ventilation in patients with mild to moderate acidosis and respiratory distress, with the aim of preventing deterioration to a point when invasive ventilation would be considered [105]. The authors of the ERS/ATS guidelines [105] suggest considering NIMV in AECOPD patients who develop acute respiratory acidosis during hospital admission, in particular when the pH is ⩽7.35, PaCO_2_ is > 45 mmHg and the respiratory rate is > 20–24 breaths/minute despite standard medical therapy [105]. The authors suggest that there is no lower limit of pH below which a trial of NIV is inappropriate even if close monitoring is needed in patients with a lower pH due to a greater risk of failure due to the possibility of a rapid endotracheal intubation [105]. Previous [106] and recent Cochrane reviews [107] confirm the effectiveness of NIMV on very severe outcome such as need for endotracheal intubation and mortality, and the magnitude of advantages appears similar for patients with mild acidosis (pH 7.30 to 7.35) versus severe (pH < 7.30), and when NIMV is applied in the ICU or in a ward setting [107].

A cornerstone study about NIMV in hospitalized AECOPD was conducted by Brochard and colleagues [99]. In a prospective, randomized study comparing NIMV plus standard treatment (oxygen until a maximal flow rate of 5 L/m by nasal prongs, in order to achieve an arterial oxygen saturation above 90%, antibiotics, bronchodilators, and corticosteroids or/and aminophylline) versus standard treatment in patients admitted to the ICU, the authors demonstrated a reduction in the need for endotracheal intubation (26% versus 74%, *p* < 0.001 in NIMV and standard therapy, respectively). Furthermore, the percentage of complications not present on admission (such as pneumonia, barotrauma, gastrointestinal hemorrhage, renal insufficiency, neurologic events, and pulmonary embolism) (16% versus 48%, *p* = 0.001), the mean length of hospital stay (23 ± 17 days versus 35 ± 33, *p* = 0.005), and the mortality rate (9.3% versus 28.5%, *p* = 0.02) were lower in the NIMV group in comparison to standard treatment [99]. The same outcomes have been demonstrated in other trials, including in patients admitted to a general respiratory ward [102, 108], or performed on patients with different etiologies of ARF other than COPD (non-COPD-related pulmonary process, neuromuscular disease, and status post-extubation) [103]. Improvements in dyspnea sensation after NIMV use have also been documented [109, 110]. Finally, the long-term survival of AECOPD patients was better in those treated with NIMV [111].

To evaluate the cost effectiveness of NIMV an incremental cost analysis per in-hospital death of 236 patients with mild to moderate respiratory acidosis (pH 7.25–7.35) due to a severe AECOPD and admitted to 14 medical wards in the United Kingdom was performed [112]. Patients receiving NIMV had a reduction in costs of £49.362 (78.741 $; 73.109 €), mainly through reduced use of ICU admission [112]. The incremental cost effectiveness ratio was -£645/death avoided (95% CI -£2310 to £386), demonstrating that NIMV is a highly cost-effective strategy [112].

Generally, NIMV requires the use of facial or nasal interfacies that are key variables for the success of treatment [113, 114]. However, a recent study demonstrated, during an episode of acute hypercapnic respiratory failure, that a new helmet may be a valid alternative to a mask in improving gas analysis, achieving a good tolerance [115]. Therefore, the sequential use of a mask and helmet diminished the incidence of failure [116]. Finally, an open mouthpiece method of ventilation, similar to traditional NIV delivered by a nasal mask, has been reported to be a useful technique, preventing further deterioration of gas exchange in AECOPD patients with mild to moderate acidosis [117]. According to strategies to improve the optimization of patient-ventilator interactions, the analysis of flow and pressure waveforms generated by ventilators can be useful [118]; optimized ventilation led to a more rapid normalization of pH at 2 h, to a significant improvement of the patient’s tolerance to ventilation at 2 h, and to a higher reduction in PaCO_2_ at 2 and 6 h [118].

Among strategies to improve the efficacy of NIMV the use of an inhaled helium/oxygen mixture (heliox) instead of an air/oxygen mixture has been proposed as adjunctive therapy to NIMV in severe AECOPD. The rationale for the use of helium is related to the low density of this element in comparison to air. Physiologically, in 10 patients with AECOPD heliox reduced the work of breathing and PCO_2_ levels without significantly changing their breathing pattern or oxygenation [119]. Although a helium/oxygen mixture improves respiratory acidosis and the respiratory rate more quickly than air/O_2_ [120], some recent trials in hypercapnic AECOPD patients found no significant effects of helium/oxygen mixture on clinical outcomes, such as the rate of NIMV failure [120–122] or intubation rate [123].

Regarding predictors of NIMV failure, the severity of hypercapnia and acidosis at admission are associated with the early failure of treatment [111, 124], while improvements in acidosis and respiratory rate after 4 h of NIMV were associated with success. Charts of risk describe the probability of failure in ARF patients treated with NIMV [125]. Very recently, the early and noninvasive ultrasound evaluation of diaphragmatic dysfunction (DD) during severe AECOPD has been proved to be reliable and accurate in identifying patients at major risk for NIMV failure and worse prognosis [126]. Patients with DD, evaluated by a change in diaphragm thickness (ΔTdi) < 20% during tidal volume, had a higher risk for NIMV failure than patients without DD (risk ratio 4.4; *p* < 0.001), and this was significantly associated with higher in-hospital and 90-day mortality rates, longer mechanical ventilation duration, higher tracheostomy rate, and longer ICU stay [126]. The accuracy to predict failure by ΔTdi < 20% was higher than baseline pH value and early change in both arterial blood pH and partial PCO_2_ following the initiation of NIV (area under the curve [AUC] was 0.84, 0.51, 0.56, and 0.54, respectively; *p* < 0.0001) [126].

### Main take home messages

NIMV represents the main treatment for hypercapnic ARF patients with associated respiratory acidosis. Although the HFNC oxygen therapy is able to reduce the level of hypercapnia, the use in clinical practice appears controversial and not clear due to very limited studies. At the moment a cautious approach appears motivated.

### Unmet needs or future clinical research

In AECOPD patients with moderate respiratory acidosis (pH between 7.35 and 7.25) prospective randomized noninferiority trials considering HFNC oxygen therapy versus NIMV are ongoing [127, 128] with the aim to evaluate physiological [128] or clinical (endotracheal intubation prevention) [127] effects. Future studies need to clarify when or not HFNC may be proposed as a valid alternative choise for hypercapnic ARF patients and when could be better to swich to NIMV; such as endotracheal intubation for NIMV, a delay in treatment with HFNC could have a worse outcome in AECOPD patient.

## Pulmonary rehabilitation

Pulmonary rehabilitation (PR) is a comprehensive non-pharmacological treatment to which COPD is most susceptible to improvements [129]. Exercise capacity and symptoms such as dyspnea and quality of life represent the main outcomes [129–132]. PR benefits COPD patients with different functional stages [133] and different phenotypes [134], the elderly [135], those with fewer or more comorbidities [136, 137], those with physical frailty [138], and even cachectic patients [139]. Historically, PR has been proposed for COPD patients in a stable phase of disease [129] and just after an AECOPD exacerbation [140], but not during AECOPD due to safety-related aspects. However, recent evidence also demonstrates the efficacy of a PR program for severe hospitalized AECOPD patients [141–147].

AECOPD reduces the physical activity of patients [148]. Hospitalization due to AECOPD leads to physical and functional impairment [149] with loss of quadriceps muscle strength [150], exercise tolerance [151] and health status [152]. The role of PR during hospitalization may thus be justified.

The first evidence on PR in respiratory acute patients was published by Trooster and colleagues in 2010 [141] and opened up new possibilities for early rehabilitation treatment [153]. The authors demonstrated, in a randomized trial on 40 severe AECOPD, that an additional resistance training program is feasible and safe during hospitalization and can prevent muscle deterioration [141]. Patients who received training showed an improvement in quadriceps force and walking capacity (documented by 6MWD [6-min walking distance]) at discharge; improvements were also documented at 1 month of follow up [141]. Similar and very recent studies on severe hospitalized AECOPD patients [142] and frail-elderly patients [143] suggest improvements in lower-limb muscle strength [142, 143], balance [143], exercise capacity [142, 143], and quality of life [142]. Feasibility studies on neuromuscular electrical stimulation (NMES) showed improvements in quadriceps muscle strength [154, 155]. Thus, all studies proposed for severe hospitalized AECOPD patients have found rehabilitative intervention to be feasible and safe [142–146, 154, 155]. At this point in time, only one randomized trial [156] aimed at reducing the readmission rate at 12 months (outcome failed) has documented a higher mortality risk in the treated group (OR 1.74, 95% CI 1.05 to 2.88, *p* = 0.03) [156]; however, the difference in mortality began > 5 months after the completion of the intervention and was not related to the early rehabilitation intervention [157].

### Main take home messages

In hospitalized AECOPD an early PR program has proved to be feasibility and safe. However, there are still few evidences to recommend this treatment for patients admitted to hospital.

### Unmet needs or future clinical research

Prospective studies are needed to identify the PR interventions providing the greatest benefits.

### Summary of recommendations or suggestions

Table 4 reports the summary of recommendations or suggestions from the GOLD document, NICE guidelines and ERS/ATS guidelines about the pharmacological and non-pharmacological treatments used in the management of a severe AECOPD.

**Table 4:** Summary of recommendations or suggestions from the GOLD document, NICE guidelines and ERS/ATS guidelines about management of severe AECOPD

In-hospital AECOPD treatments	GOLD document	NICE guidelines	ERS/ATS guidelines
Bronchodilators	SABA with or without short-acting anticholinergics are the initial bronchodilators recommended. It is recommended that patients do not receive continuous nebulization, but use the MDI inhaler. It is recommended continuining long-acting bronchodilators or starting as soon as possible	Increased doses of short-acting bronchodilators are suggested. Both nebulisers and hand-held inhalers can be used to administer inhaled therapy. Patients should be changed to hand-held inhalers as soon as their condition has stabilised	It is not reported
Steroids	Prednisone 40 mg per day for 5 days is recommended. Therapy with oral prednisolone is equally effective to intravenous administration	Prednisolone 30 mg orally should be prescribed for 7 to 14 days	Suggested oral administration (conditional recommendation, low quality of evidence)
Antibiotics	They should be given in patients who have: all three symptoms (increase in dyspnea, sputum volume, and sputum purulence); increased sputum purulence; required mechanical ventilation. The recommended length of therapy is 5 to 7 days	Antibiotics should be used to treat exacerbations of COPD associated with a history of more purulent sputum	It is not reported for hospitalized patients with AECOPD
Oxygen therapy	If necessary, oxygen should be given to keep the SaO_2_ within the individualised target range	Supplemental oxygen should be titrated to improve hypoxemia, with a target SaO_2_ of 88 to 92%	It is not reported
HFNC	In patients with hypoxemic ARF it may be an alternative to standard oxygen therapy or NIMV. There is a need for well-designed, randomized, multicenter trials to study the effects of HFNC in hypoxemic/hypercapnic ARF.	It is not reported	It is not reported
NIMV	It is indicated in patients with respiratory acidosis or severe dyspnoea with clinical signs suggestive of respiratory muscle fatigue, increased work of breathing, or persistent hypoxemia despite supplemental oxygen therapy	It should be used as the treatment of choice for persistent hypercapnic ventilatory failure during exacerbations despite optimal medical therapy	It is recommended for patients with acute or acute-on-chronic hypercapnic respiratory failure (strong recommendation, low quality of evidence)
Pulmonary rehabilitation	It is not reported for hospitalized patients with AECOPD	It is not reported for hospitalized patients with AECOPD	It is suggested not initiating during hospitalisation (conditional recommendation, very low quality of evidence)

Reported from references [1, 9, 10]

Abbreviations: *SABA* indicates short-acting β_2_ agonists, *MDI* metered-dose inhaler, *HFNC* high flow nasal cannulae oxygen therapy, *NIMV* non-invasive mechanical ventilation, *SaO*_*2*_ oxygen saturation, *ARF* acute respiratory failure

## Non-conventional drugs

Some non-conventional approaches have been proposed in severe AECOPD [158–160].

In a randomized double-blinded clinical trial the effect of nebulized furosemide (40 mg) as an adjunct to conventional treatment has been tested in the emergency department on 100 AECOPD patients [158]. Changes in dyspnea perception, FEV_1_, arterial blood gas variables, blood pressure, breathing frequency, and heart rate at baseline and at 1 h were the study measurements. In both groups (nebulized furosemide and saline) all measured variables were improved at 1 h, although FEV_1_, dyspnea score, PaO_2,_ pH, blood pressure, breathing frequency, heart rate, improved significantly more in the furosemide group. Patients presenting a lower FEV_1_ on admission had more benefit from furosemide than those who had a higher baseline FEV_1_ [158].

Another randomized, placebo-control, double-blind, crossover trial [159] enrolling 24 AECOPD patients has tested the effect of magnesium sulfate (1.5 g intravenous for 20 min). FEV_1_ was measured after 15, 30, and 45 min of magnesium sulfate or placebo administration and after a dose of 400 μg of salbutamol. In term of FEV_1_ after 15, 30, and 45 min no differences were found between groups, while the FEV_1_ measured after salbutamol found significantly greater increases in the magnesium sulfate group in comparison to placebo (+ 0.185 L and + 0.081 L; *p* = 0.004, respectively) [159]. In conclusion, the magnesium sulfate has no bronchodilatation effect in AECOPD patients, although it enhances the bronchodilating effect of salbutamol [159].

The last randomized double-blind, placebo-controlled, parallel group trial has evaluated the effect of oral zileuton (a 5-lipoxygenase inhibitor having an effect on leukotrienes) at the dose of 2400 mg daily for 14 days [160]. The primary outcome was the length of hospital stay, while the secondary ones were treatment failure and biomarkers of leukotriene production. There was no difference in the length of hospital stay (3.75 days versus 3.86 days, *p* = 0.39 for zileuton and placebo groups, respectively) and treatment failure (23% versus 27%, *p* = 0.63 for zileuton and placebo groups, respectively), despite a decline in urinary leukotrienes levels in the zileuton-treated group at 24 and 72 h [160]. Due to the slow recruitment the study has been stopped before the enrollement target and for this reason the sample size analysed may have been insufficient for the meaningful.

## Conclusion

Several treatments exist for severe AECOPD patients requiring hospitalization. However, some treatments lack strong evidence. Strong evidence currently exists for steroids and NIMV, in patients admitted with a hypercapnic acute respiratory failure and respiratory acidosis. HFNC oxygen therapy needs further evidences to be suggested. Concerning the use of antibiotics, while many benefits have been reported for ICU patients, there is a lack of evidence on the preferred drug and optimal duration of treatment for hospitalized patients. Early rehabilitation, if associated with standard treatment of patients, should be proposed due to its feasibility and safety: we should apply the rule that “more is better’”. There are currently few options involving new drugs or new applications of existing drugs.

## Abbreviations

AECOPD: Acute exacerbation of chronic obstructive pulmonary disease
ARF: Acute respiratory failure
COPD: Chronic obstructive pulmonary disease
CRP: C-reactive protein
DD: Diaphragmatic dysfunction
DPI: Dry powder inhalers
FEV_1_: Forced expiratory volume in the 1^st^ second
FVC: Forced vital capacity
GNEB: Gram-negative enteric bacilli
HFNC: High flow nasal cannulae
ICU: Intensive care unit
IQR: Interquartile range
ITT: Intention-to-treat
LABA: Long-acting β_2_ agonists
MDI: Metered-dose inhaler
MRCT: Microorganisms resistant to conventional treatment
MSCT: Microorganisms sensitive to conventional treatment
NIMV: Non-invasive mechanical ventilation
OR: Odds ratio
PaO_2_: Partial arterial oxygen pressure
PP: Per-protocol
SABA: Short-acting β_2_ agonists
